# Disc Test for Detecting Staphylococcus aureus Strains Producing Type A and Type C β-Lactamases

**DOI:** 10.1128/spectrum.00220-23

**Published:** 2023-07-06

**Authors:** C. R. Robert George, Monica M. Lahra, Thanh Nguyen, Barrie Gatus

**Affiliations:** a NSW Health Pathology, Microbiology, John Hunter Hospital, Newcastle, Australia; b NSW Health Pathology, Microbiology, Prince of Wales Hospital, Randwick, Australia; c School of Medical Sciences, Faculty of Medicine, the University of New South Wales, Sydney, Australia; The University of North Carolina at Chapel Hill

**Keywords:** cefazolin inoculum effect, disc test, *Staphylococcus aureus*

## Abstract

Staphylococcus aureus can produce β-lactamases capable of hydrolyzing penicillins and first-generation cephalosporins. The propensity of type A and type C β-lactamase-producing S. aureus (TAPSA and TCPSA) to hydrolyze cefazolin at a high inoculum is termed the cefazolin inoculum effect (CIE). Strains with a CIE have a theoretical risk of causing treatment failure and are unable to be detected routinely by most laboratories. We developed a high-performing yet straightforward β-lactamase disc test that identifies and differentiates both TAPSA and TCPSA and is suitable for routine diagnostic laboratory workflows. Clinical isolates of S. aureus resistant to penicillin were identified, and their *blaZ* genes were sequenced. MICs were determined at low and high inocula (5 × 10^5^ CFU/mL and 5 × 10^7^ CFU/mL), and isolates demonstrating a CIE were characterized. A semimechanistic model was established to describe differential hydrolysis patterns, and candidate models were iteratively assessed using area-under-the-curve analysis from competitor receiver operating characteristic (ROC) curves. Biomarker thresholds were derived from Youdon index-derived optimal cutoff values. Genetic analysis of 99 isolates identified 26 TAPSA isolates and 45 TCPSA isolates. The model best differentiating TAPSA from non-TAPSA utilized cefazolin-to-cephalothin ratio analysis (sensitivity, 96.2%; specificity, 98.6%). The model best differentiating TCPSA from non-TCPSA incorporated cefazolin, cephalothin, and oxacillin (sensitivity, 88.6%; specificity, 96.6%). TAPSA and TCPSA can be differentiated using three antibiotic discs on a single agar plate. The test has potential value in typing the β-lactamase type from isolates from patients that are candidates for or have failed cefazolin therapy.

**IMPORTANCE** The key significance of this article is that it details a straightforward method of performing a disc test that can differentiate Staphylococcus aureus isolates that are likely to be associated with a cefazolin inoculum effect and theoretical risk of cefazolin treatment failure from isolates that are less likely to be associated with a cefazolin inoculum effect.

## INTRODUCTION

Staphylococcus aureus is a well-established pathogen that has developed numerous antimicrobial resistance strategies. β-Lactam-based drugs are a mainstay of treatment but can be rendered ineffective via β-lactamase production or penicillin-binding-protein modification (i.e., methicillin-resistant S. aureus [MRSA]). Four classes of staphylococcal β-lactamases are described (A, B, C, and D), with expression being either constitutive or inducible (1–3) and genomic assessment indicating at least 29 allotypes and 43 substitutions (4). Each class demonstrates characteristic hydrolysis profiles (5), with type A β-lactamase-producing S. aureus (TAPSA) and, to a lesser extent, type C β-lactamase-producing S. aureus (TCPSA) being noted for their propensity to hydrolyze cefazolin, particularly at high inocula (5–8), as opposed to type B and type D β-lactamase-producing S. aureus (TBPSA, and TDPSA, respectively). This effect, described as the cefazolin inoculum effect (CIE), has been variously described as either an MIC of ≥16 μg/mL at high inocula and ≤8 μg/mL at low inocula (9) or a ≥4-fold increase in MIC at high inocula versus low inocula (6, 10). The concern is that under conditions associated with a higher inoculum of organisms (e.g., abscesses, endocarditis, osteomyelitis, pneumonia, and septic arthritis), the higher burden of organisms could trigger a CIE, resulting in treatment failure (9).

There is potential clinical value in a simple test that can identify S. aureus strains that are likely to cause a CIE. It is widely recognized that TAPSA can hydrolyze cefazolin at high inocula *in vitro* (11, 12). *In vivo*, experimental animal models demonstrate higher mortality associated with cephazolin therapy of TAPSA in endocarditis in rabbits (13) and in intraperitoneal infection in mice (14) and in attempts to reduce the number of CFU per gram of vegetation in rats (15). Cefazolin use in prophylaxis has been identified as a risk factor for postsurgical TAPSA wound infection (11, 16), and treatment failures have long been reported for humans with infective endocarditis infected with S. aureus (12, 17–20). An increased 30-day mortality was noted in an assessment of patients from Argentina (21). Unfortunately, the lack of high-quality and controlled clinical trials has long, and repeatedly, been recognized (7, 22–24). As a result, the clinical relevance of the CIE remains unresolved (6, 8, 10, 25). Nonetheless, a link between persistent bacteremia and CIE has been demonstrated (6). Further, cefazolin use (with or without a demonstrated CIE) has been linked with treatment failure, albeit without statistical significance (6, 9, 10, 26). Other studies have reported that cefazolin has efficacy similar to or better than that of alternative agents (22–24, 26, 27), although uncertainty remains, as all clinical studies published to date demonstrate key weaknesses that limit their interpretability (28). Consequently, the potential importance of the CIE under conditions associated with high inocula of S. aureus has been widely noted (6, 9, 10). Given that cefazolin may have treatment advantages compared with other agents (8, 24, 25) and is recommended for β-lactam-allergic patients with methicillin-sensitive S. aureus endocarditis (25, 29), there is potential value in a simple test that determines whether isolates from such patients could be associated with an inoculum effect. Additionally, although a CIE caused by TCPSA has been noted previously, often at lower rates (6, 8, 9), the CIE is more frequently associated with and studied in the context of TAPSA. Consequently, while there is value in a disc test that identifies TAPSA, given the potential but less understood role of TCPSA, being able to identify and differentiate TCPSA would be informative.

Standardized testing systems (e.g., Clinical and Laboratory Standards Institute [CLSI] and European Committee on Antimicrobial Susceptibility Testing [EUCAST] systems) have historically faced challenges detecting staphylococcal β-lactamase producers; both methods recommend careful assessment of penicillin disc zone edges (30). Chromogenic cephalosporinase tests (e.g., nitrocefin) are inferior for detecting β-lactamase production and were removed from CLSI and EUCAST guidelines in 2012 (31). Of note, neither CLSI or EUCAST offers a convenient method of differentiating TAPSA even when suspected. Despite the potential to hydrolyze cefazolin, TAPSA typically tests susceptible by routine broth or disc methods of susceptibility testing. Consequently, β-lactamase type determination requires more elaborate methods, which often fit poorly with workflows in routine diagnostic laboratories, given limitations of cost, time, and expertise.

For diagnostic purposes, no disc test is available for predicting an S. aureus isolate’s β-lactamase type, with differentiation of type being historically challenging (32, 33). A colorimetric test has recently been proposed, with reasonable performance against TAPSA but lesser performance against TCPSA (34). Richmond’s antiserum, which was originally used to type isolates, is depleted, and, to our knowledge, no replacement exists. Nucleic acid testing (NAT) of the *blaZ* gene followed by bioinformatic assessment to predict the serotype presents a high-sensitivity and -specificity solution (for example, see references 9 and 35). However, NAT is not widely performed, given the need for bioinformatic analysis, as well as cost- and turnaround time-based considerations. We are not aware of any commercial NATs that differentiate staphylococcal β-lactamases. Enzyme kinetic studies of hydrolysis profiles have been described (5). However, such studies are resource intensive and require specialist training and interpretation, which precludes their routine use in most diagnostic laboratories. MIC analysis using current breakpoints is considered less reliable than disc testing for detecting penicillinase production (36). MIC testing of low and high isolate concentrations (e.g., 5 × 10^5^ CFU/mL and 5 × 10^7^ CFU/mL) against cefazolin identifies strains producing a CIE. However, the method is time-consuming, and given the high- versus low-level expression of β-lactamase from different strains, it remains challenging to predict a strain’s β-lactamase type. While modified nitrocefin tests may have some value (for example, see reference 34), EUCAST warns that chromogenic cephalosporin-based β-lactamase tests do not reliably detect staphylococcal penicillinase (36).

Given that cefazolin represents a therapeutic mainstay for S. aureus in many regions and for numerous indications (37, 38), there remains a role for a screening test that can be included in a diagnostic laboratory’s workflow. Ideally, such a test would be sensitive, specific, and inexpensive, have a rapid turnaround time, and be easily performed. In this study, we describe a disc test (the *blaZ*-R disc test) that identifies and differentiates β-lactamase resistance for both TAPSA and TCPSA and can be performed using three antibiotic discs on a single agar plate.

## RESULTS

### Circulating staphylococcal types.

Sequence analysis revealed the presence of all four described classes of β-lactamase circulating in the hospitals tested, with TCPSA being most prevalent (*n *= 45, i.e., 45% of clinical isolates), followed by TBPSA (*n *= 27, i.e., 27% of isolates) and then TAPSA (*n *= 26, i.e., 26% of isolates). Only a single isolate of TDPSA was isolated (i.e., 1% of isolates).

Three clinical isolates were excluded from analysis due to overlapping peaks in the sequence data that prevented β-lactamase type determination. The Sanger sequence of the type D β-lactamase-producing control isolate FAR 10 was similarly uninterpretable due to overlapping peaks. Given that bioinformatic analysis of published control sequences for all β-lactamase types (i.e., including type D β-lactamase) was successful at predicting β-lactamase type, a clinical isolate which had a *blaZ* sequence consistent with type D β-lactamase production was retained.

### MICs and the inoculum effect.

Cefazolin, cephalothin, cephalexin, and flucloxacillin showed variable hydrolysis profiles depending on β-lactamase type (Table 1). Among the test strains, TAPSA demonstrated the greatest ability to hydrolyze cefazolin (up to six doubling dilutions), followed by TCPSA (up to four doubling dilutions). In contrast, test strains of TCPSA demonstrated a greater inoculum effect for cephalothin than TAPSA (TCPSA, maximum, three doubling dilutions; TAPSA, maximum, two doubling dilutions). Type B β-lactamase-producing S. aureus showed little CIE (maximum, two doubling dilutions). Given that only a single isolate TDPSA was detected, there was limited opportunity to assess its potential to cause a CIE. During data processing, it was noted that one isolate failed to grow in the control well, and this isolate was excluded from analysis.

**TABLE 1:** Distribution of cefazolin, cephalothin, cephalexin, and flucloxacillin MICs for isolates tested at 5 × 10^7^ CFU/mL and the distribution of MIC fold differences for isolates tested at 5 × 10^5^ CFU/mL versus 5 × 10^7^ CFU/mL^*a*^

Antibiotic	MIC	No. of strains					DD (F)	No. of strains				
		A	B	C	D	All		A	B	C	D	All
Cefazolin	0.5		4			4	0 (0)	1	5	1		7
	1	1	11	5		17	1 (2)	1	13	8		22
	2	6^*b*^	11^*c*^	12		29	2 (4)	*11* ^*b*^	*9* ^*c*^ ^,^ ^*d*^	*29*	*1*	*50*
	4	6	1^*d*^	21 ^*e*^	1	29	3 (8)			*2* ^*e*^		*2*
	8	1		5		6	4 (16)	*4*		*5*		*9*
	16	**5**		**2**		**7**	5 (32)	*7* ^*f*^				*7*
	32	**6** ^*f*^				**6**	6 (64)	*2*				*2*
	64	**1**				**1**	7 (128)	—^*g*^				
	128	—^*g*^					8 (256)					

Cephalothin	0.25	1	1			2	0 (0)	4	2	1		7
	0.5	7^*b*^	4	3		14	1 (2)	11 ^*b*^	14	9		34
	1	12	13	6		31	2 (4)	11^*f*^	9^*c*^	27 ^*e*^	1	48
	2	5^*f*^	7^*c*^	27 ^*e*^	1	40	3 (8)	—^*g*^	2^*d*^	8		10
	4	1^*g*^	2^*d*^	9		12	4 (16)					

Cephalexin	4		1			1	0 (0)	1	4			5
	8	2	6	1		9	1 (2)	3^*b*^	10 ^*c*^	12	1	26
	16	7^*b*^	17 ^*c*^ ^,^ ^*d*^	15		40	2 (4)	11	12^*d*^	27 ^*e*^		50
	32	11	3	27 ^*e*^		41	3 (8)	10^*f*^	1	6		17
	64	4^*f*^		2		6	4 (16)	1				1
	128	1				1	5 (32)	—^*g*^				
	256	1^*g*^				1	6 (64)					

Flucloxacillin	0.25	2	1	1		4	0 (0)	1	3	2		6
	0.5	5	16	8		29	1 (2)	11	19 ^*c*^ ^,^ ^*d*^	24	1	55
	1	14 ^*b*^	10^*c*^^,^^*d*^	29 ^*e*^	1	54	2 (4)	12 ^*b*^ ^,^ ^*g*^	5	19^*e*^		36
	2	4^*f*^^,^^*g*^		7		11	3 (8)	2^*f*^				2
	4	1				1	4 (16)					
Total		26	27	45	1	99		26	27	45	1	99

a TAPSA demonstrated the greater cefazolin hydrolysis at a high inoculum but lesser hydrolysis of cephalothin versus TBPSA and TCPSA. Isolates of TCPSA also demonstrated a CIE. Boldface values indicate isolates meeting the CIE criteria of an MIC of ≥16 μg/mL at a high inoculum and ≤8 μg/mL at a low inoculum (9); italicized values indicate isolates with a ≥4-fold increase in MIC at a high inoculum versus a low inoculum (6, 10). Underlining indicates the median value for each type. MIC was determined for isolates inoculated at 5 × 10^7^ CFU/mL. F, fold difference between an isolate’s MIC determined at 5 × 10^5^ CFU/mL versus 5 × 10^7^ CFU/mL; DD, number of doubling dilutions between an isolate’s MIC as determined at 5 × 10^5^ CFU/mL versus 5 × 10^7^ CFU/mL.

b ATCC 29213 (type A).

c 22260 (type B).

d ST79/741 (type B).

e 3804 (type C).

f NCTC 9789 (type A).

g PC1 (type A).

Using the criteria of a CIE being an MIC of ≥16 μg/mL at a high inoculum and ≤8 μg/mL at a low inoculum (9), 12 of 26 TAPSA strains (46%) demonstrated a CIE, compared with 2 of 45 TCPSA strains (4%). Overall, using this definition, 14/99 (14.1%) isolates demonstrated a CIE. Using the less widely used criterion of a ≥4-fold increase in MIC at high inocula versus low inocula (6, 10), 24 of 26 TAPSA strains (i.e., 92%) demonstrated a CIE compared with 36 of 45 TCPSA strains (80%). Using the latter criterion, the sole TDPSA isolate and 9 of 27 TBPSA isolates (33%) also demonstrated a CIE, although only isolates of TAPSA and TCPSA demonstrated a ≥8-fold increase in MIC. Overall, using this definition, 70/99 (70.7%) isolates demonstrated a CIE.

A Pearson correlation of changes in doubling dilution observed in test strains between antibiotic pairs showed a positive correlation for the majority of pairs, with the remainder being near zero (Table 2). The strength of the correlation varied by β-lactamase type and antibiotic combination, suggesting that while increased hydrolysis for one antimicrobial correlates with increased hydrolysis for a second antimicrobial under circumstances of an increased inoculum, the β-lactamase class modifies the response. This phenomenon was similarly seen in the control strains.

**TABLE 2:** *R* values derived from Pearson’s correlation of doubling dilution values for different antibiotic combination subsets by β-lactamase type^*a*^

Antibiotic	*R* for β-lactamase type								
	CZL			CLT			CLX		
	A	B	C	A	B	C	A	B	C
FLU	0.38	0.21	0.29	0.5	0.11	0.41	0.72	−0.02	0.4
CLX	0.49	−0.03	0.31	0.63	0.06	0.18			
CLT	0.6	0.69	0.59						

a Numbers of strains tested were 26 (type A), 27 (type B) and 45 (type C). Type D was not tested, as only a single strain was present. The majority of combinations showed a positive correlation, with the remainder being near zero. The strength of the correlation varies by antibiotic and β-lactamase, suggesting that while increased hydrolysis for one antimicrobial correlates with increased hydrolysis for a second antimicrobial under circumstances of an increased inoculum, the β-lactamase class modifies the response. CZL, cefazolin; CLT, cephalothin; CLX, cephalexin; FLU, flucloxacillin.

### Zone sizes.

Radial zone sizes varied by β-lactamase and disc type (Table 3). For each antimicrobial agent, there was overlap between disc zone sizes for all β-lactamase types, indicating that no disc zone range derived from an individual disc solely differentiated between types.

**TABLE 3:** Disc zone sizes for test strains stratified by antibiotic and β-lactamase^*a*^

Antibiotic	Zone size (mm)	No. of strains				
		A	B	C	D	All
Cefazolin (30 μg)	8 ≤ *x *< 9	3		1		4
	9 ≤ *x *< 10	7		6		13
	10 ≤ *x *< 11	9	1	23	1	34
	11 ≤ *x *< 12	5	7	13		25
	12 ≤ *x *< 13		11	1		12
	13 ≤ *x *< 14	2	7	1		10
	14 ≤ *x *< 15					
	15 ≤ *x *< 16					
	16 ≤ *x *< 17		1			1

Cephalothin (30 μg)	8 ≤ *x *< 9			1		1
	9 ≤ *x *< 10			4		4
	10 ≤ *x *< 11	1	5	18	1	25
	11 ≤ *x *< 12	9	10	18		37
	12 ≤ *x *< 13	7	8	3		18
	13 ≤ *x *< 14	6	2			8
	14 ≤ *x *< 15		1	1		2
	15 ≤ *x *< 16	3				3
	16 ≤ *x *< 17		1			1

Cephalexin (100 μg)	7 ≤ *x *< 8	1				1
	8 ≤ *x *< 9					
	9 ≤ *x *< 10	1				1
	10 ≤ *x *< 11	5	1	10		16
	11 ≤ *x *< 12	12	3	18		33
	12 ≤ *x *< 13	4	9	10		24
	13 ≤ *x *< 14	1	7	3		11
	14 ≤ *x *< 15	1	5	1		7
	15 ≤ *x *< 16		1			1

Oxacillin (1 μg)	3 ≤ *x *< 4			1		1
	4 ≤ *x *< 5	2				2
	5 ≤ *x *< 6	2		9		11
	6 ≤ *x *< 7	9	1	18		28
	7 ≤ *x *< 8	7	7	15	1	30
	8 ≤ *x *< 9	3	13	1		17
	9 ≤ *x *< 10	3	5	1		9
	10 ≤ *x *< 11					
	11 ≤ *x *< 12					
	12 ≤ *x *< 13		1			1

Penicillin (1 U)	0 ≤ *x *< 1	10	7	29		46
	1 ≤ *x *< 2	12	12	13	1	38
	2 ≤ *x *< 3	2	4	2		8
	3 ≤ *x *< 4		2			2
	4 ≤ *x *< 5	2	1	1		4
	5 ≤ *x *< 6		1			1

Cefoxitin (10 μg)	6 ≤ *x *< 7		1			1
	7 ≤ *x *< 8	5	2	8		15
	8 ≤ *x *< 9	14	12	17		43
	9 ≤ *x *< 10	6	10	16	1	33
	10 ≤ *x *< 11		2	2		4
	11 ≤ *x *< 12	1		2		3

a TAPSA and TCPSA had smaller cefazolin and oxacillin disc zones than TBPSA. TCPSA had a smaller cephalothin disc zone than TAPSA and TBPSA. Median values are underlined.

For differentiation of TAPSA from TCPSA, cefazolin (30 μg), cephalexin (100 μg), oxacillin (1 μg), and cefoxitin (10 μg) all demonstrated similar disc zone sizes (i.e., median zone sizes within 0 to 1 mm). However, differing distributions were seen for cephalothin (30 μg) for (median zone size difference, 2 to 3 mm), with TAPSA having demonstrably larger zones than TCPSA. For the differentiation of TCPSA from TBPSA, cefazolin (30 μg) and oxacillin (1 μg) demonstrated the greatest difference in zone size (median zone size difference, 2 to 3 mm), with TBPSA demonstrating larger zones. Cephalothin (30 μg) and cephalexin (30 μg) demonstrated smaller zone size differences (1- to 2-mm median zone size difference), with TBPSA again demonstrating larger zones. Zone sizes for cefoxitin (10 μg) showed no discernible difference. These observations supported the possibility of using disc zone ratios to differentiate types.

A Pearson correlation of changes in disc zone sizes versus changes in MIC doubling dilution stratified by β-lactamase type for cephalexin, cefazolin, and cephalothin demonstrated that, in general, strains that demonstrate a larger inoculum effect between low and high inocula are associated with smaller zones for doubling dilution increases in cephalothin and cefazolin, with the effect being less pronounced for cephalexin (Table 4).

**TABLE 4:** *R* values derived from a Pearson’s correlation analysis of MIC doubling dilution against disc zone sizes stratified by β-lactamase type for cephalexin, cefazolin, and cephalothin^*a*^

Antibiotic for disc zone size measurement	*R* for doubling dilution change from MIC testing for β-lactamase type								
	CZL			CLT			CLX		
	A	B	C	A	B	C	A	B	C
CZL	−0.27	−0.37	−0.36	−0.46	−0.45	−0.27	−0.01	−0.07	0.04
CLT	−0.32	−0.3	−0.27	−0.48	−0.44	−0.26	0.08	−0.05	0.05
CLX	−0.51	−0.29	−0.15	−0.59	−0.32	−0.11	−0.28	0.11	−0.03

a Strains that demonstrated a larger inoculum effect between low and high inocula were typically associated with smaller zones, particularly for doubling dilution increases in cephalothin and cefazolin. Type D was not tested, as only a single strain was present. CZL, cefazolin; CLT, cephalothin; CLX, cephalexin.

### Candidate models.

**FIG 1:**
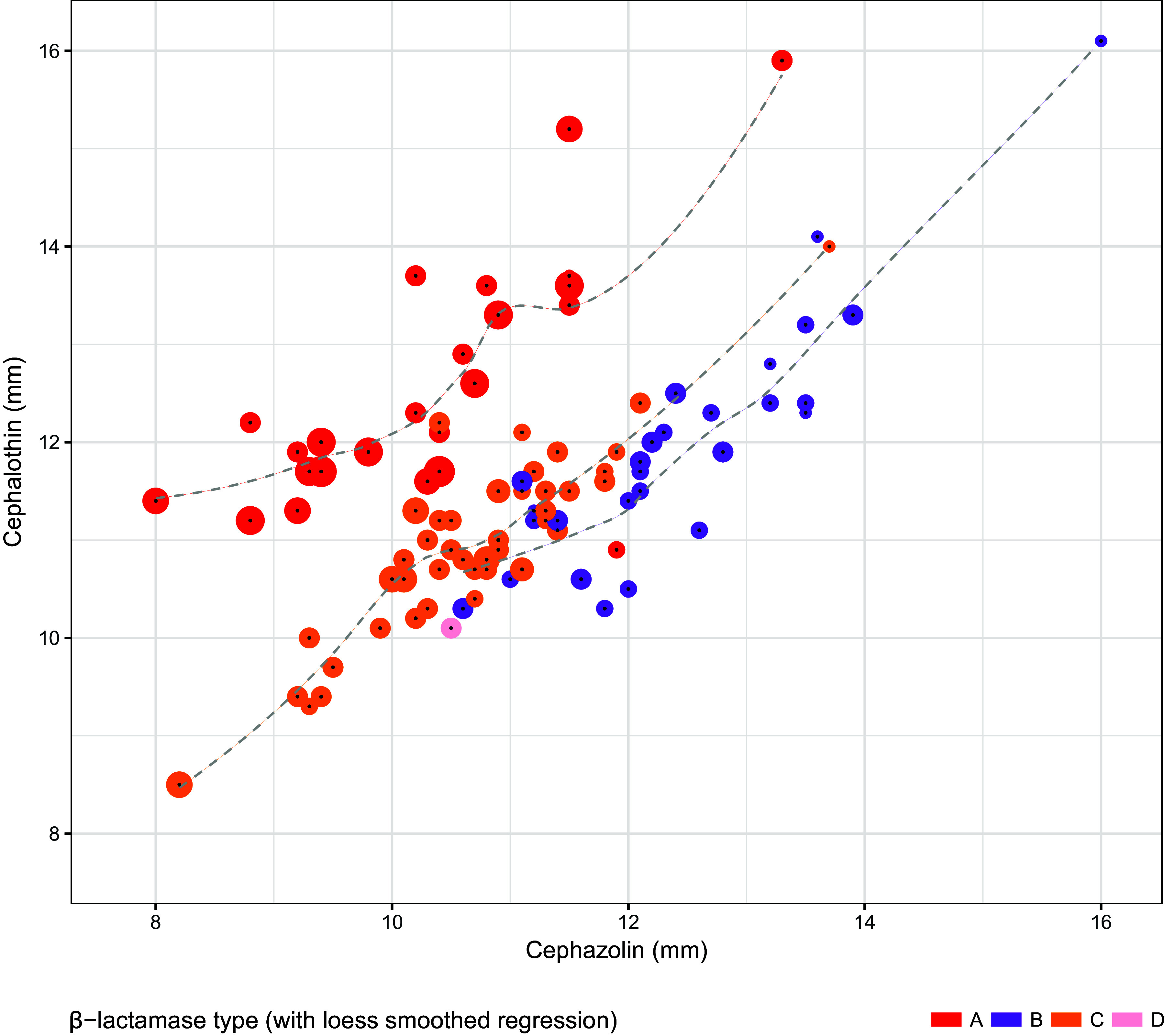
Separation of type A β-lactamase-producing Staphylococcus aureus from other β-lactamase producers when comparing cephalothin (30 μg) disc zone sizes against cefazolin (30 μg) disc zone sizes. Point sizes reflect the number of doubling dilutions associated with the size of the cefazolin inoculum effect (range, 0 to 6).

**FIG 2:**
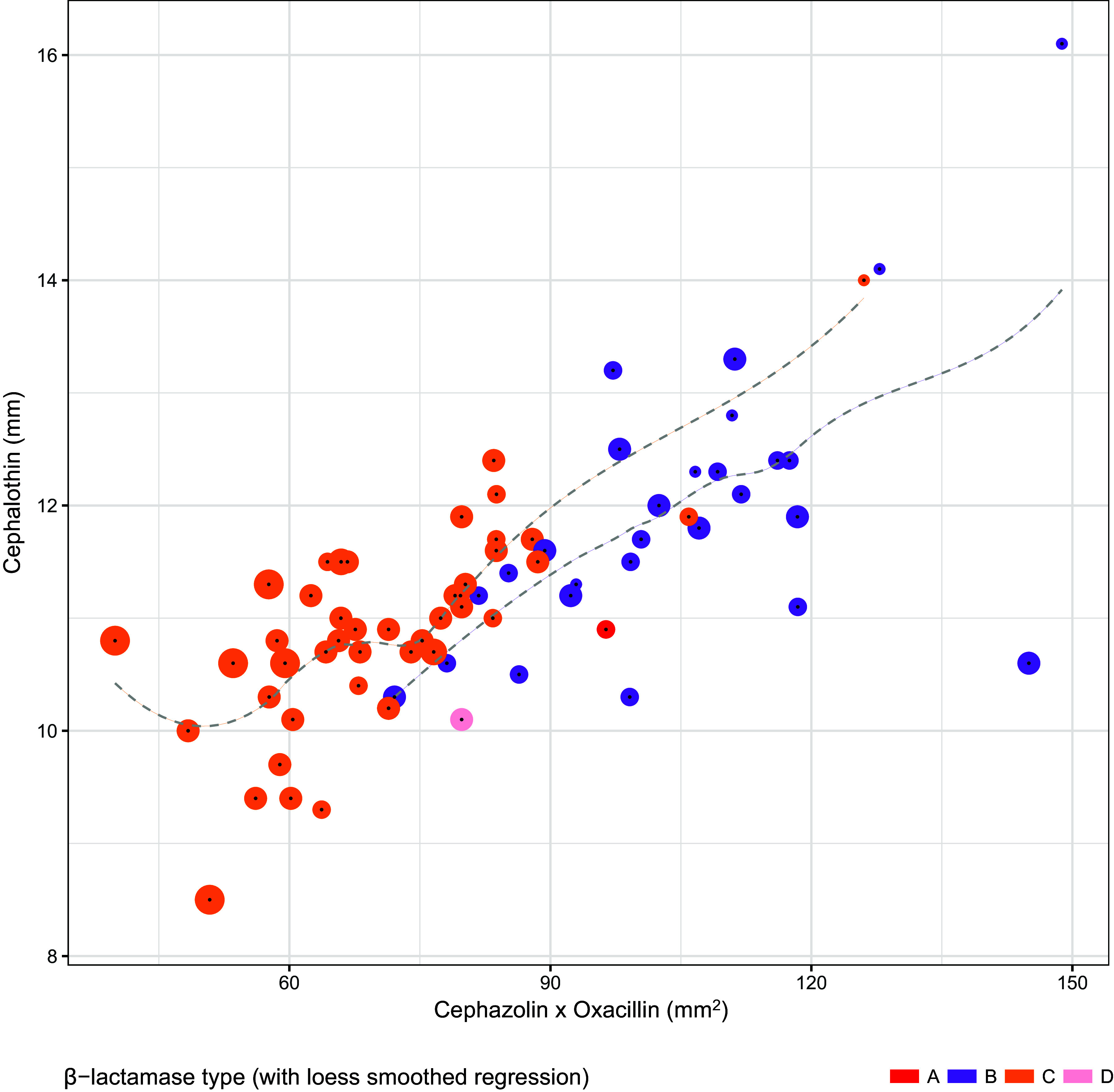
Distribution of type C β-lactamase-producing Staphylococcus aureus versus other β-lactamase producers when comparing cephalothin (30 μg) disc zone sizes against cefazolin (30 μg) × oxacillin (1 μg) disc zone sizes after predicted type A β-lactamase-producing S. aureus isolates are removed. Point sizes reflect the number of doubling dilutions associated with the cefazolin inoculum effect (range, 0 to 4).

Candidate models were established for TAPSA and TCPSA using the semimechanistic nested approach described below. Regarding TAPSA, univariate analysis of disc sizes revealed the greatest performance of cephalothin and cefazolin disc zone size (Table 4). This conformed with disc zone data that demonstrated that, unlike other types, TAPSA is typically associated with both a small zone for cefazolin and a large zone for cephalothin. Assessment of antimicrobial disc zone sizes demonstrated separation of TAPSA from other β-lactamase producers when cefazolin was assessed against cephalothin (Fig. 1) (see Table 5 for the comparative performance candidate models). Regarding TCPSA (once identified TAPSA isolates were excluded), a combination of cephalothin, cefazolin, and oxacillin showed the best performance. Disc zone data supported the observation that TCPSA isolates frequently (but not universally) have a smaller disc zone for oxacillin. Assessment of cefazolin and oxacillin as a function of cephalothin demonstrated a degree of separation of TCPSA from remaining β-lactamase producers (Fig. 2) (see Table 6 for the comparative performance of candidate models). For each β-lactamase type, the inoculum effect varied in magnitude across the range of zone sizes measured.

**TABLE 5:** Candidate models for TAPSA^*a*^

Iteration and model^*b*^	No.	Step from no.	Sn	Sp	PPV	NPV	ACC	PLR	NLR	AUC	Step rank	Final rank	Rationale^*c*^
1. Univariate analysis													
−CLT	1		0.846	0.658	0.468	0.923	0.707	2.471	0.234	0.806	1	4	E
CZL	2		0.577	0.74	0.441	0.831	0.697	2.217	0.572	0.696	2	6	E
CL	3		0.76	0.529	0.365	0.86	0.589	1.612	0.454	0.628	3	7	E
FOX	4		0.731	0.466	0.328	0.829	0.535	1.368	0.578	0.590	4	8	E
OX	5		0.462	0.685	0.343	0.781	0.626	1.465	0.786	0.542	5	9	E
−P	6		0.615	0.493	0.302	0.783	0.525	1.214	0.78	0.506	6	10	E
2. Bivariate ratio analysis													
CZL/CLT	7	2/1	0.961	0.986	0.962	0.986	0.980	70.192	0.040	0.962	1	1	D, M
CL/CLT	8	3/1	0.880	0.829	0.647	0.951	0.842	5.133	0.145	0.872	2	3	D, M
OX/CLT	9	5/1	0.808	0.685	0.477	0.909	0.717	2.564	0.281	0.764	4	5	D
3. Multiplicative analysis													
(CZL × CL)/(CLT × CLT)	10	7 × 8	0.92	0.914	0.793	0.970	0.916	10.733	0.088	0.939	2	2	
(CL/CLT) × −CLT	11	8 × 1	0.24	0.471	0.140	0.635	0.411	0.454	1.612	0.373	11	11	
(CZL/CLT) × −CLT	12	7 × 1	0.423	0.26	0.169	0.559	0.303	0.572	2.217	0.304	12	12	

a Univariate assessments were performed for all disc zone sizes. Bivariate ratios were then determined for disc combinations that disc testing or MIC testing indicated might be of value given differing distributions identified between β-lactamase types. The third iterative stage multiplied combinations of the three top-performing candidates from previous steps to assess if additional information was provided. The top-performing model was TAPSA ~ CZL/CLT. Sn, sensitivity; Sp, specificity; PPV, positive predictive value; NPV, negative predictive value; ACC, accuracy; PLR, positive likelihood ratio; NLR, negative likelihood ratio.

b CZL, cefazolin; CLT, cephalothin; CL, cephalexin; OX, oxacillin; P, penicillin; FOX, cefoxitin.

c E, empirical assessment (including ongoing empirical assessment of the top three performing univariate candidates based on AUC and any derivatives); D, assessment based on observed differences in distribution of disc testing results; M, assessment based on observed differences in distribution of MIC testing results.

**TABLE 6:** Candidate models for TCPSA^*a*^

Iteration and model^*b*^	No.	Step from no.	Sn	Sp	PPV	NPV	ACC	PLR	NLR	AUC	Step rank	Final rank	Rationale^*c*^
1. Univariate analysis													
OX	1		0.841	0.897	0.925	0.788	0.863	8.129	0.177	0.922	1	4	E
CZL	2		0.886	0.759	0.848	0.815	0.836	3.672	0.15	0.891	2	5	E
CL	3		0.643	0.929	0.931	0.634	0.757	9	0.385	0.839	3	7	E
P	4		0.75	0.724	0.805	0.656	0.74	2.719	0.345	0.736	4	10	E
CLT	5		0.818	0.552	0.735	0.667	0.712	1.825	0.33	0.727	5	11	E
FOX	6		0.477	0.448	0.568	0.361	0.466	0.865	1.166	0.514	6	13	E
2. Bivariate ratio analysis													
CZL/CLT	7	2/5	0.909	0.793	0.870	0.852	0.863	4.394	0.115	0.882	3	6	D, M
OX/CL	8	1/3	0.619	0.964	0.963	0.628	0.757	17.333	0.395	0.828	5	8	D
OX/CLT	9	1/5	0.727	0.793	0.842	0.657	0.753	3.515	0.344	0.817	6	9	D
CZL/CL	10	2/3	0.833	0.393	0.673	0.611	0.657	1.372	0.424	0.611	9	12	D, M
3. Multiplicative analysis													
OX × CZL/CLT	11	1 × 7	0.886	0.966	0.975	0.848	0.918	25.705	0.118	0.951	1	1	
OX × CZL	12	1 × 2	0.909	0.862	0.909	0.862	0.890	6.591	0.105	0.929	2	2	
CZL × CZL/CLT	13	2 × 7	0.977	0.759	0.86	0.957	0.890	4.049	0.030	0.927	3	3	

a Univariate assessments were performed for all disc zone sizes. Bivariate ratios were then determined for disc combinations that disc testing or MIC testing indicated might be of value given differing distributions identified between β-lactamase types. The third iterative stage multiplied combinations of the three top-performing candidates from previous steps to assess if additional information was provided. The top-performing model was TCPSA ~ OX × CZL/CLT. Sn, sensitivity; Sp, specificity; PPV, positive predictive value; NPV, negative predictive value; ACC, accuracy; PLR, positive likelihood ratio; NLR, negative likelihood ratio.

b CZL, cefazolin; CLT, cephalothin; CL, cephalexin; OX, oxacillin; P, penicillin; FOX, cefoxitin.

c E, empirical assessment (including ongoing empirical assessment of the top three performing univariate candidates based on AUC and any derivatives); D, assessment based on observed differences in distribution of disc testing results; M, assessment based on observed differences in distribution of MIC testing results.

**FIG 3:**
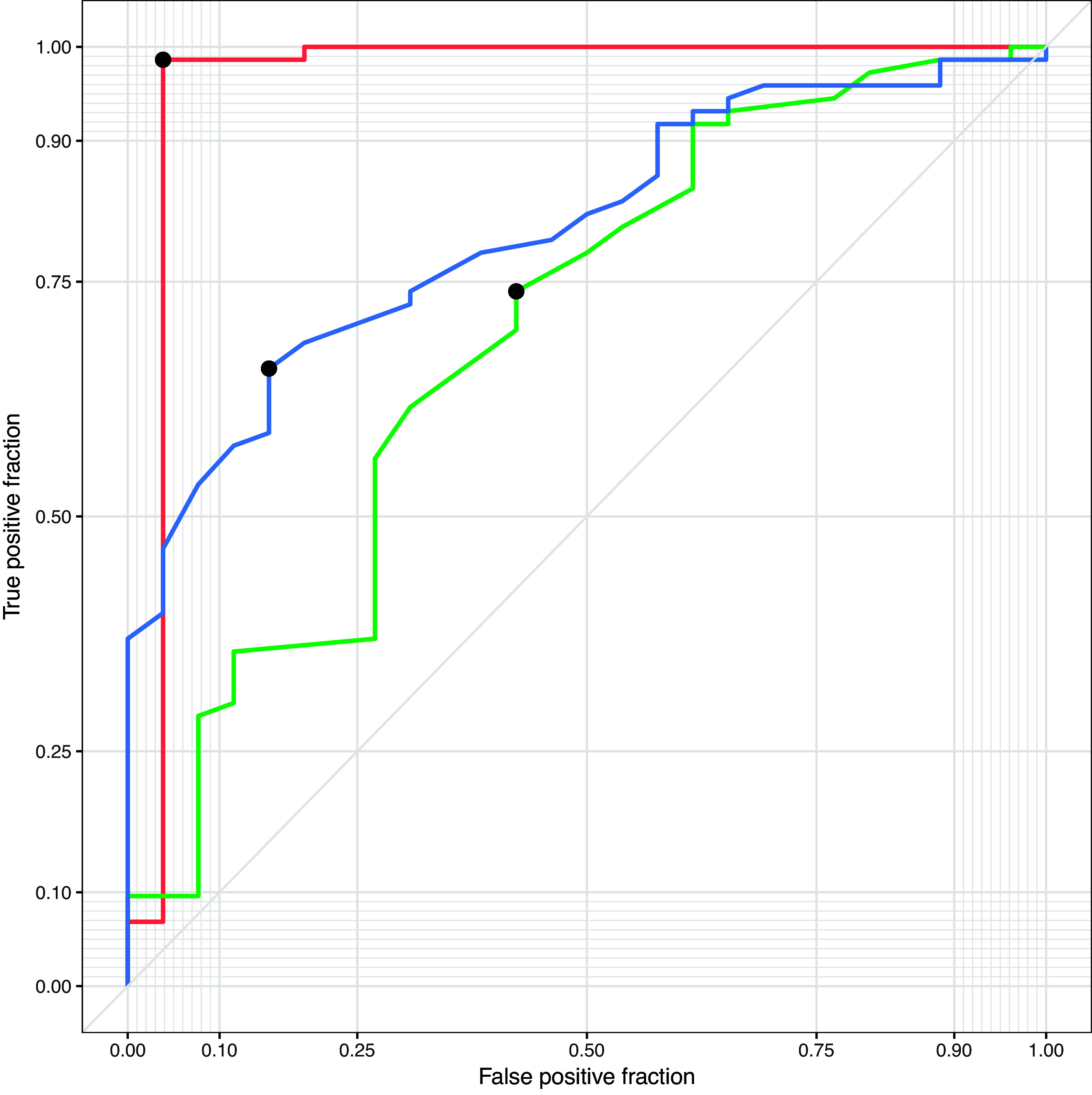
Receiver operating characteristic curve with optimum cutoff values (black dots) for the detection of type A β-lactamase-producing Staphylococcus aureus using the *blaZ*-R disc test (red; threshold = 0.896, AUC = 0.962), which outperformed cefazolin (30 μg) disc testing (green; threshold = 10.45 mm, AUC = 0.696) and cephalothin (30 μg) disc testing (blue; threshold = 11.55 mm, AUC = 0.806).

Based on observed hydrolysis profiles and published data, the optimal criterion defined for TAPSA prediction was

(1) $$x_{\text{A}}|x_{\text{A}}\in X_{\text{A}+\text{B}+\text{C}+\text{D}}\text{, }x_{\text{A}}=\left(\text{CZL/CLT}\;\leq \;0.896\right)$$

where *x*_A_ is test values predictive of TAPSA, *X*_A+B+C+D_ represents test values for all β-lactamase-producing S. aureus isolates that are not MRSA, CZL represents cefazolin zone size, CLT represents cephalothin zone size, and the value 0.896 is derived from a Youdon optimal cutoff value. Test characteristics for detecting TAPSA were as follows: sensitivity, 0.962; specificity, 0.986; area under the receiver operating characteristic curve (AUC ROC), 0.962 (Fig. 3); positive predictive value, 0.962; negative predictive value, 0.986; accuracy, 0.98; positive likelihood ratio, 70.192; and negative likelihood ratio, 0.039.

**FIG 4:**
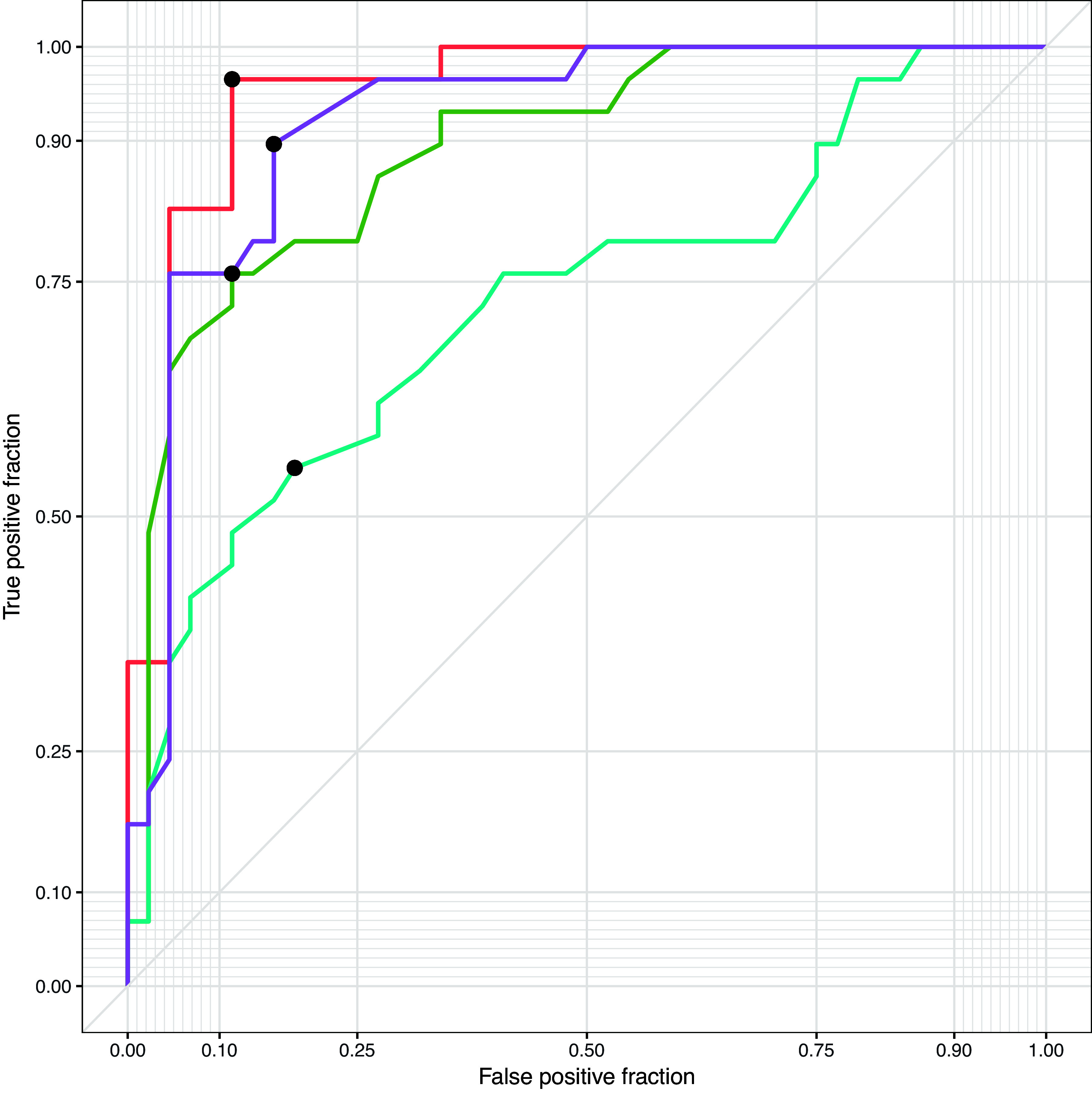
Receiver operating characteristic curve with optimum cutoff values (black dots) for the detection of type C β-lactamase-producing Staphylococcus aureus using the *blaZ*-R disc test (red; threshold = 7.26 mm, AUC = 0.951), which outperformed cephalothin (30 μg) disc testing (blue; threshold = 11.55 mm, AUC = 0.727), cefazolin (30 μg) disc testing (green; threshold = 11.55 mm, AUC = 0.891), and oxacillin (1 μg) disc testing (purple; threshold = 7.15 mm, AUC = 0.922).

Based on observed hydrolysis profiles and published data, the optimal criterion defined for TCPSA prediction was

(2) $$x_{\text{C}}|x_{\text{C}}\in X_{\text{A}+\text{B}+\text{C}+\text{D}}\wedge x_{\text{C}}\notin x_{\text{A}},\;x_{\text{C}}=\left(\text{OX}\times \text{CZL/CLT}\;\leq \;7.26\text{ mm}\right)$$

where *x*_C_ is test values predictive of TCPSA, OX represents oxacillin zone size, and the value of 7.26 mm is derived from a Youdon optimal cutoff value. Test characteristics for detecting TCPSA were as follows: sensitivity, 0.886; specificity, 0.966; AUC ROC, 0.951 (Fig. 4); positive predictive value, 0.975; negative predictive value, 0.848; accuracy, 0.918; positive likelihood ratio, 25.705; and negative likelihood ratio, 0.118.

### Model evaluation.

**FIG 5:**
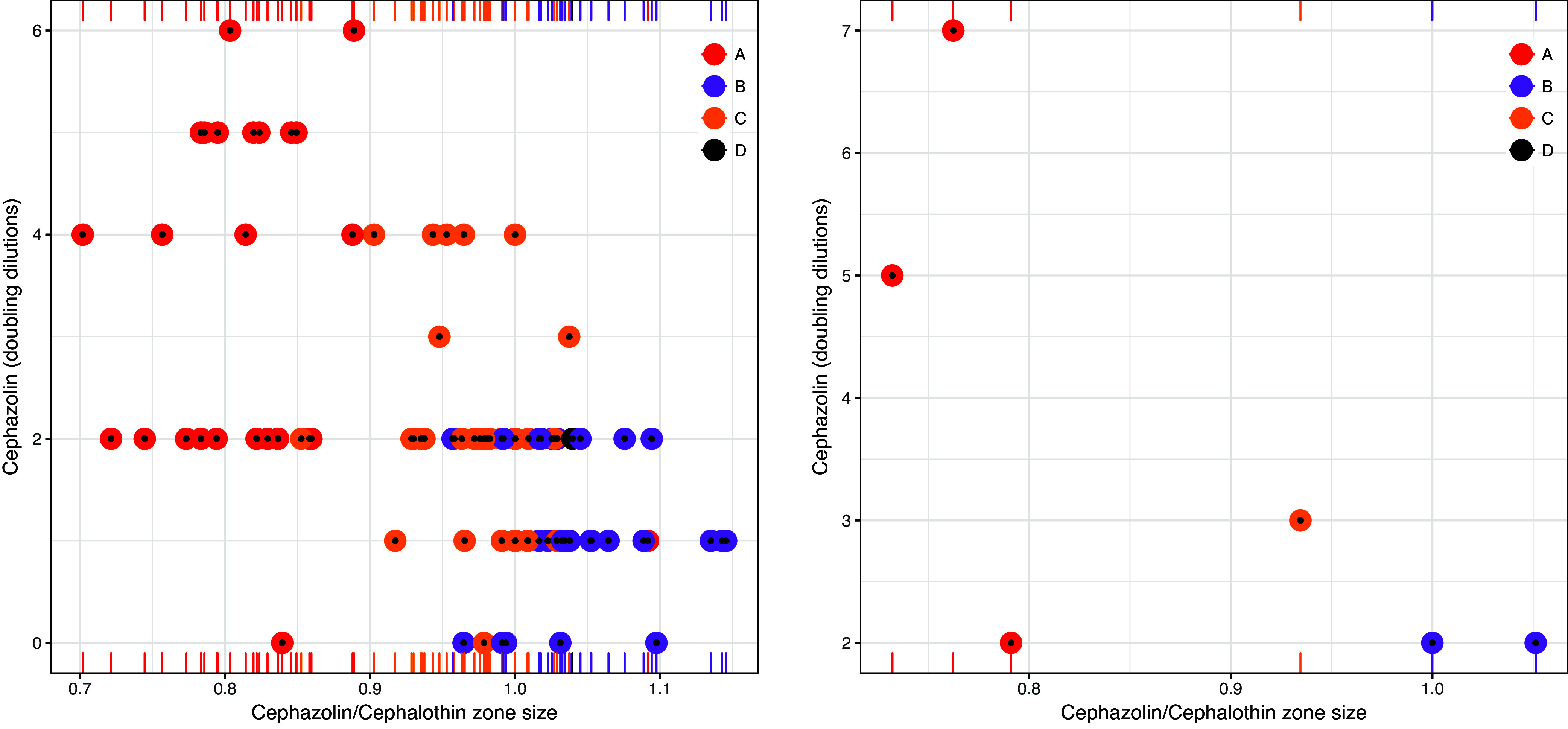
Performance of the TAPSA model for identifying clinical isolates (left) and control isolates (right) based on a cefazolin-to-cephalothin ratio of 0.896. All control isolates were correctly predicted.

**FIG 6:**
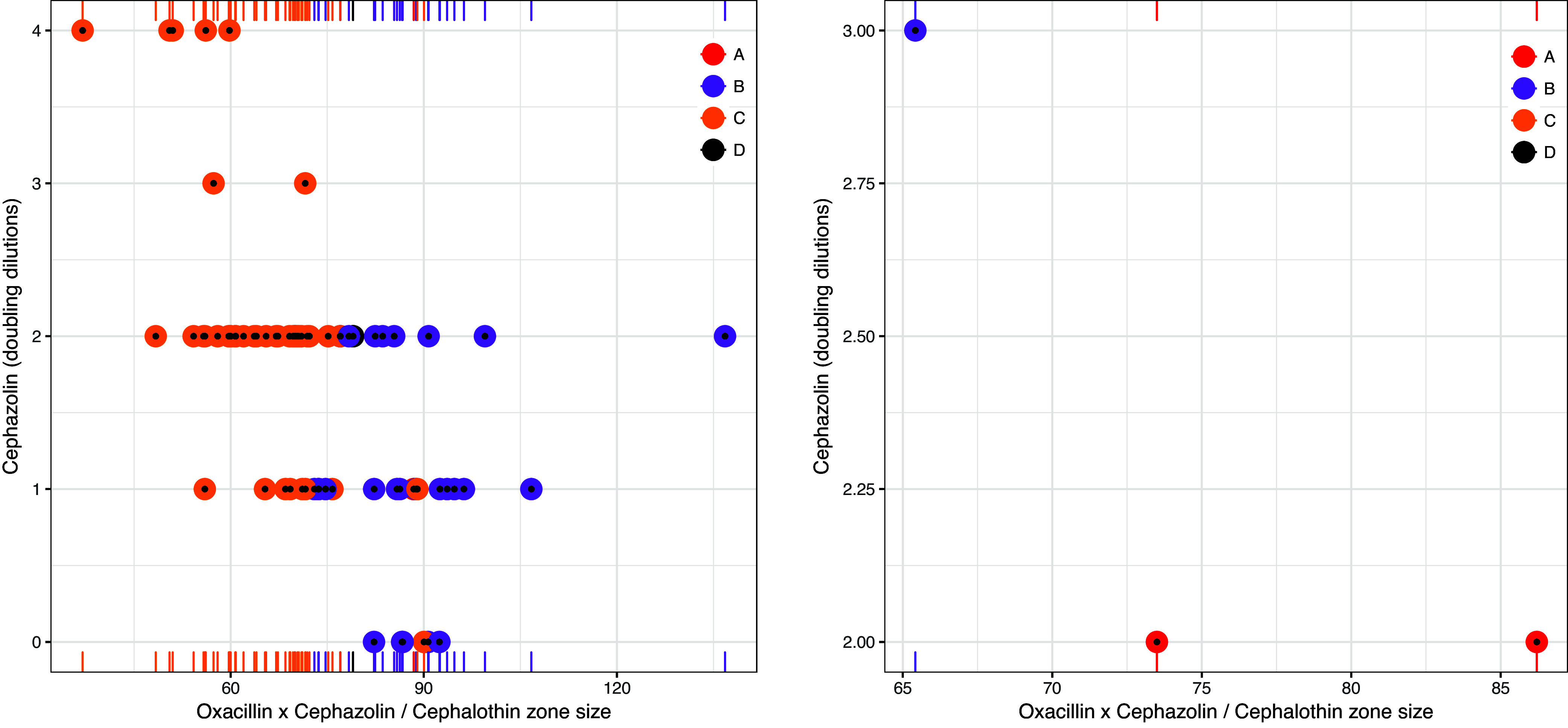
Performance of the TCPSA model for identifying clinical isolates (left) and control isolates (right) based on a cefazolin-to-cephalothin ratio of 7.26. All control isolates were correctly predicted.

The TAPSA and TCPSA models were applied to the control strains to evaluate their performance. The TAPSA model successfully predicted the identity of the TAPSA control strains (Fig. 5), while the TCPSA model then successfully predicted the identity of the TCPSA control strains (Fig. 6).

## DISCUSSION

There is merit in a simple test that can inexpensively and accurately identify β-lactamase producers capable of hydrolyzing cefazolin. The *in vitro* evidence presented here supports the idea that β-lactamases produced by TAPSA preferentially hydrolyze cefazolin, and animal models support this finding (13–15). While a significant association between treatment failure and CIE has not been established in the clinical studies performed to date (6, 8–10, 39–42), it is noted that studies have suffered from weaknesses that limit their interpretability (28, 43); clinical studies relating to the CIE and treatment failure must therefore be interpreted with caution due to such issues as general quality, sample size, heterogeneity, publication bias, and low rate of deep-seated infections. Similarly, factors that limit the interpretability of studies assessing treatment equivalence of cefazolin include study design (26, 27) and the fact that the studies did not specifically assess for the presence of the CIE or *blaZ* type (22, 23, 26, 27). The tests proposed here are therefore clinically relevant, as they provide an opportunity for laboratories to assess the likelihood that a strain is TAPSA (or potentially TCPSA).

Beyond epidemiological interest, knowing the β-lactamase type of an S. aureus strain may be useful for several reasons. The test could be used to screen S. aureus isolates from patients diagnosed with conditions such as infective endocarditis. If the strain is a non-TAPSA, non-TCPSA strain, the likelihood of a CIE is lower. An alternative application involves the retrospective application of the test to patients with failed cefazolin therapy (e.g., recurrent S. aureus bacteremia). Identifying a TBPSA strain would effectively rule out the CIE as a cause of failure.

We found that 26% of strains were TAPSA, which is consistent with other studies which have demonstrated values between 15 and 34% (28). The strain most frequently detected was TCPSA (45% of isolates), which is in agreement with other studies (9). Using the definition of the CIE published by Nannini et al. (an MIC of ≥16 μg/mL at high inocula and ≤8 μg/mL at low inocula) (9), 14.1% of circulating strains demonstrated the CIE; 42.3% of TAPSA isolates demonstrated a CIE, with 50% of strains demonstrating an MIC increase of 16- to 64-fold. Conversely, only 4.4% of TCPSA isolates demonstrated a CIE, with the maximum MIC increase being 16-fold. Using the alternative definition of CIE (an MIC increase of ≥4-fold between low and high inocula), we found that 70.7% of circulating strains demonstrated a CIE, including several TBPSA isolates (not usually associated with the CIE) and 24 of 26 (92.3%) TAPSA strains. This discordance in the definition of CIE may partially explain the large variation in reported global rates, with some studies reporting that up to 94.1% of TAPSA isolates demonstrate a CIE (6). The observation is of significance for studies attempting to assess the relevance of the CIE to treatment outcomes. At least five studies of treatment outcome used the definition of an MIC of ≥16 μg/mL at a high inoculum and ≤8 μg/mL at a low inoculum (8, 9, 21, 39, 44), while at least three used the definition of a ≥4-fold increase in cefazolin MIC between low and high inocula (6, 10, 40). We found that the latter definition resulted in a substantial increase in the number of isolates defined as having a CIE. Investigators assessing the possibility of links between the CIE and treatment failure should assess whether the use of a less specific metric of CIE risks falsely rejecting possible associations. Consequently, we suggest that investigators consider both definitions when assessing the clinical implications of the CIE.

**FIG 7:**
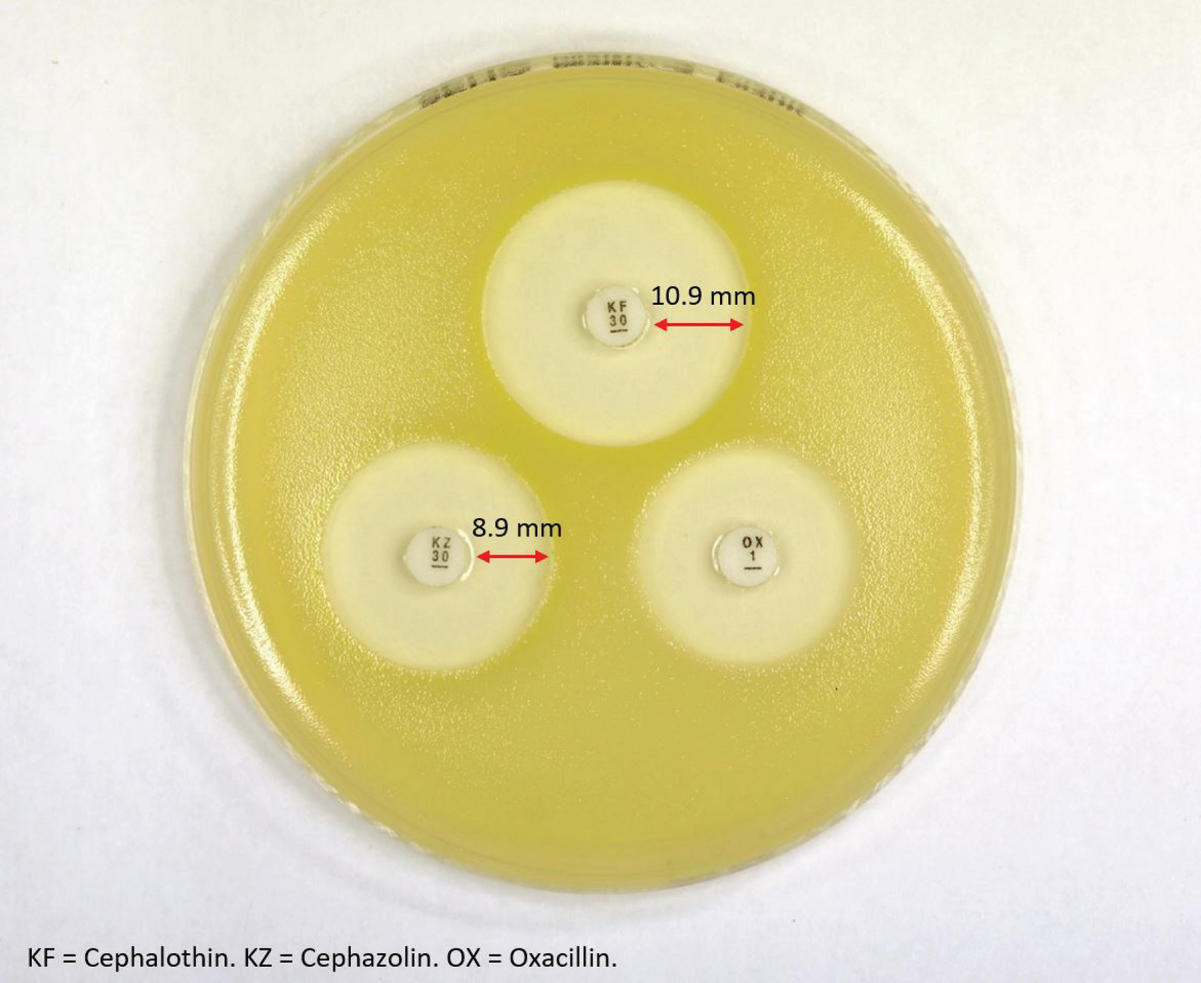
Example of the disc *blaZ*-R test setup using the control strain ATCC 29213 (a known TAPSA strain). For this example, KZ = 8.9 mm, KF = 10.9 mm, and KZ/KF = 8.9/10.9 = 0.817. Given that this value is less than the optimal cutoff of 0.896, the isolate is predicted to be TAPSA, and no further assessment for TCPSA (using the OX disc) is required.

We recommend using and interpreting the *blaZ*-R disc test as follows. (i) Inoculate and incubate plates as described. (ii) Using Vernier calipers, measure the zone size from the disc edge to the edge of confluent growth for cefazolin (variable, CZL), cephalothin (variable CLT), and oxacillin (variable, OX). (iii) Calculate CZL/CLT. Isolates with values of ≤0.896 are predicted to be TAPSA, and no further disc analysis is required. If the value is >0.896, proceed to step 4, as the isolate is unlikely to be TAPSA. (iv) Calculate OX × CZL/CLT. Isolates with values of ≤7.26 mm are predicted to be TCPSA. If the value is >7.26, the isolate is less likely to be TCPSA (e.g., it may be TBPSA). As a demonstration, Fig. 7 provides a photographic demonstration of the *blaZ*-R test using the strain ATCC 29213, which is known to produce type A β-lactamase.

This study has several unique features. It provides the first assessment of a disc test capable of identifying TAPSA (and TCPSA) which are associated with the CIE. Previous methods of assessment have included NAT, enzyme kinetics, or serology testing, with the latter no longer being available. Second, it is the first study to assess the rate of TAPSA and CIE in an Australian context. Of note, the sample of isolates tested had a rate of TAPSA roughly equivalent to rates described elsewhere, although further interpretation is hampered by differing definitions of the CIE. Third, this is the first attempt to mechanistically relate the S. aureus CIE with disc testing. Indeed, while previous studies have identified relationships between β-lactamase type and β-lactam hydrolysis profiles, to our knowledge, this is the first to consider biomarker performance of CIE from a mechanistic perspective.

Although the described tests performed well, various limitations should be noted. First, our testing was based on a small number of historic strains from one geographic region. While model evaluation was performed against reference strains and demonstrated reproducible performance, further evaluation of previously typed strains, as well as strains from other regions, is warranted. Additionally, we detected only a single type D β-lactamase-producing strain, and although this was accurately predicted as non-TAPSA and non-TCPSA by the *blaZ*-R disc test, further assessment is required. Additionally, further analysis, including of test reproducibility, repeatability, and ruggedness would be beneficial (45). Second, our assessment of β-lactamase type is based on genetic markers, given that serotyping solution is no longer available. We note that an isolate incorrectly predicted by the *blaZ*-R disc test as TCPSA was identified by Sanger sequencing and bioinformatic analysis as TAPSA, although chromatographic analysis revealed a secondary peak that was consistent with TCPSA. Further work is required regarding the possibility, importance, and nature of β-lactamase expression in isolates potentially carrying *blaZ* gene variants. Third, the performance of the *blaZ*-R disc test for identifying TCPSA is dependent on careful exclusion of TAPSA. To provide the most realistic estimates of performance, our calculation of the *blaZ*-R disc test’s performance in predicting TCPSA included isolates that were initially misclassified during the TAPSA detection step. Fourth, while the *blaZ*-R test’s semimechanistic model is designed to identify β-lactamase type reliably, it is not designed to provide an assessment of the probability that the predicted type will have a CIE. Additional modeling work that further explores disc-based solutions to this dilemma would be beneficial. Fifth, the study did not assess the workflow considerations associated with introducing the *blaZ*-R test. It is essential that laboratories considering this tool consider it in the context of their patient population, preexisting workflows, intended use, and financial circumstances. Finally, this study did not assess the clinical implications of the CIE, for which further studies are required that are sufficiently powered, control for selection bias, include deep-seated infections, and adequately define CIE and *blaZ* type and prevalence.

The *blaZ*-R disc test permits an accurate, rapid, straightforward, and low-cost solution to predicting an S. aureus isolate’s β-lactamase type. Further work is required to assess the characteristics of β-lactamase-producing S. aureus and to determine the true risks associated with cefazolin and the CIE.

## MATERIALS AND METHODS

### Sample collection and susceptibility profiling.

Ninety-nine consecutive clinical isolates of S. aureus were collected between May and June 2013 from three tertiary hospitals in New South Wales, Australia (The Prince of Wales Hospital, Sydney Children’s Hospital, and The Royal Hospital for Women). Isolates from all sample types were reviewed, cultured on blood agar (Oxoid), and identified using matrix-assisted laser desorption–ionization time of flight (MALDI-TOF) mass spectrometry (Bruker). Isolates that tested resistant to penicillin (0.5 U) but susceptible to cefoxitin (10 μg; i.e., methicillin-susceptible, penicillin-resistant S. aureus) using calibrated dichotomous sensitivity (CDS) criteria were selected (46). Care was taken to ensure purity via serial passaging and phenotypic assessment.

### Control isolates.

Published control isolates were obtained for each of the four described β-lactamase types (A to D) (3, 5, 6, 32, 47): for type A, strain PC1(pI254), a known high-level β-lactamase producer, strain ATCC 29213, a known low-level β-lactamase producer, and strain NCTC 9789; for type B, strain 22260 and strain ST79/741; for type C, strain 3804; and for type D, strain FAR 10. Additionally, published *blaZ* sequences for strains producing each β-lactamase type (A to D) were used to verify sequence interpretation (47) (GenBank accession numbers NG_052065.1 and AF086644.1).

### Amplification and sequence analysis.

To determine each control and test isolate’s β-lactamase type, nucleic acids were extracted from isolates for nucleic acid testing using PrepMan Ultra (Applied Biosystems). The *blaZ* gene was amplified with forward primer 5′-CAAAGATGATATAGTTGCTTATTC-3′ and reverse primer 5′-CATATGTTATTGCTTGCACCAC-3′ to produce a 355-bp product as previously published (9). Sanger sequencing was performed by the Australian Genome Research Facility (AGRF) using capillary electrophoresis. Sequences were analyzed using R 3.4.0 (packages sangerseqR 1.12.0 and DECIPHER 2.4.0). Chromatograms were developed for forward and reverse sequences, and primary and secondary base calls were made. Consensus sequences were determined for each strain. A programmatic function was developed to characterize sequence types. Nucleotide substitutions at positions 128 and 216 were analyzed to determine if the resultant amino acids were concordant with published structures for β-lactamase types A, B, C, and D. Threonine at position 128 and serine at position 216 predict TAPSA, lysine at position 128 and asparagine at position 216 predict TBPSA, threonine at position 128 and asparagine at position 216 predict TCPSA, and alanine at position 128 and serine at position 216 predict TDPSA (9, 48).

### MIC determination.

All S. aureus control and test strains were assessed to determine if a CIE existed. This permitted us to assess whether TAPSA circulating in the study population demonstrated a CIE and also to determine the relative importance of other β-lactamase types (e.g., type C) regarding a CIE. MICs were determined for isolates using microtiter plates containing Mueller-Hinton broth with serial dilutions of reconstituted flucloxacillin, cefazolin, cephalothin, and cephalexin and inoculated at both low and high concentrations (5 × 10^5^ CFU/mL and 5 × 10^7^ CFU/mL). Plates were incubated at 35 to 37°C in ambient air. Low- and high-inoculum MICs were determined at 24 h through visual inspection. Two methods of characterizing a CIE were used, given the variations in the literature. The first method involved identifying isolates that had a cefazolin MIC of ≥16 μg/mL at the high inoculum and a cefazolin MIC of ≤8 μg/mL at the low inoculum (9). The second method involved assessing the fold change in MIC between low- and high-inoculum MICs to determine which strains had a ≥4-fold MIC difference between low and high inocula (6, 10).

### Disc test assessment.

Disc testing was performed for cefazolin (30 μg; Oxoid), cephalothin (30 μg; Oxoid), oxacillin (1 μg; Oxoid), cefoxitin (10 μg; Oxoid), penicillin (0.5 U; Oxoid), and cephalexin (100 μg; Oxoid) for all isolates using a CDS-based method of inoculum preparation (46). Additional exploratory testing was performed with higher-potency cefazolin discs (80 μg and 200 μg) and higher-potency cephalothin discs (80 μg and 200 μg). Zone sizes were calculated using Vernier calipers by measuring the shortest distance between the disc edge to the edge of confluent growth. Disc potencies and the selection of antibiotic types for testing were based on the availability of commercial preparations. Disc zones with noncircular or unclear zone sizes (e.g., hazy borders) were retested, and zones considered repeatedly unreliable were excluded.

### Model derivation.

Semimechanistic models were established using set theory to establish algebraic sets that provided a formulaic description of parameters descriptive of β-lactamase type and that could be easily applied in a laboratory setting. While the precise mechanisms driving the CIE are still not fully understood (49), it was possible to model β-lactamase type as a function of disc zone size by considering both *a priori* information, including biologically plausible and published hydrolysis profiles (5, 9, 12, 14, 32, 33, 48, 50), and experimental evidence from MIC and disc testing. Consideration of how disc zone sizes should be analyzed to identify specific β-lactamase types was based on the assessment of factors associated with β-lactam hydrolysis, laboratory testing, and comparative model assessment and evaluation.

### (i) β-Lactam hydrolysis considerations.

Differences in hydrolysis (*h*) relate to the amount (*a*) of β-lactamase produced (7, 14), secondary to differential genetic expression (*e*) of *blaZ* (e.g., variations in the *blaI* repressor gene [51]), and the number (*n*) of *blaZ* genes expressed (e.g., due to plasmid expression in TAPSA, TCPSA, and TDPSA [10]). Differences in hydrolysis (*h*) are also influenced by the β-lactamase type’s hydrolysis profile (*p*) (7, 10, 47), including the possible effects of intratype genetic polymorphisms (35), and the organism count (*c*) (e.g., due to inoculum and experimental conditions). Dysfunctional *agr* loci have been positively correlated with the CIE (49), but given that the mechanism remains unknown, this correlation cannot be mechanistically modeled. Consequently, for a given β-lactamase and antibiotic combination, the known relationships relating to hydrolysis can be generalized as *h* ~ *nepc*.

### (ii) Laboratory testing considerations.

Differences in β-lactam hydrolysis (*h*) affect the MIC and alter disc zone size (*z*). Increasing *h* reduces *z*, with the degree of effect influenced by experimental factors (*t*), such as agar depth, antibiotic diffusion through the agar, and antibiotic disc potency. Hence, *h* ~ *t* × (1/*z*) ~ *t*/*z*. Consequently, the disc zone size (*z*) for any β-lactamase type and antibiotic combination can be generalized as *t*/*nepc*.

### (iii) Comparative model construction, assessment, and evaluation.

Given the above-stated relationship, disc zone sizes (*z*) may be used individually or in combination to differentiate β-lactamase types as a biomarker. Assessment of biomarker performance can be iteratively undertaken using receiver operating characteristic (ROC) curves and area under the curve (AUC) assessments. Where possible, testing of antibiotic combinations should be based on biologically and mechanistically meaningful associations (i.e., *a priori* knowledge).

Hydrolysis ratios of two (or more) antibiotics have been used previously to predict β-lactamase type (33, 47). Considering disc zone sizes for two antimicrobial agents (*z_1_* and *z_2_*), one can calculate

$$\frac{z_{1}}{z_{2}}=\frac{t_{1}}{n_{1}e_{1}p_{1}c_{1}}/\frac{t_{2}}{n_{2}e_{2}p_{2}c_{2}}=\frac{t_{1}}{n_{1}e_{1}p_{1}c_{1}}\times \frac{n_{2}e_{2}p_{2}c_{2}}{t_{2}}=\frac{t_{1}n_{2}e_{2}p_{2}c_{2}}{t_{2}n_{1}e_{1}p_{1}c_{1}}$$

The method permits β-lactamase type to be isolated by cancelling out factors such as plasmid count, β-lactamase expression, and testing-related factors such as organism count, accounting for variable zone sizes and MICs. Specifically, for a given strain, *n*_1_ = *n*_2_, *e*_1_ = *e*_2_ under standardized experimental conditions (e.g., temperature, duration, atmosphere, and agar selection), organism count *c*_1_ = *c*_2_, and *t*_1_/*t*_2_ becomes constant for any antibiotic combination (denoted *k*). Hence, *z*_1_/*z*_2_ = *k*(*p*_2_/*p*_1_).

A nested approach to selecting specific β-lactamase types can be employed whereby a primary model can initially identify (and therefore permit exclusion of) a given type. The nested secondary model then selects from the remaining isolates those that match a secondary profile (e.g., TCPSA). This increases the pretest probability in the second-round model construction. The performance of resultant biomarker cutoff values can be evaluated by blindly applying them to new strains, such as control strains that represent well-characterized β-lactamase types, to determine if they provide an accurate and reproducible prediction.

Using this basis, competitor combinations of disc zone size and indexes derived from ratios of disc zone sizes were iteratively compared using receiver operating characteristic curves, with the area under the curve being compared at each step. The selection of variables (and combination of variables) for testing was based on a combination of empirical assessment (e.g., univariate disc zone sizes for all tested discs) and the assessment of results from disc testing and MIC testing data. For the first iterative step, all discs were assessed from an empirical standpoint. For the second iterative step (i.e., bivariate ratio analysis), combinations of discs and MICs were considered based on the characteristics of their distribution. Specifically, ratios were established for discs or MICs that showed markedly different distributions based on their median value and range between (i) TAPSA and TCPSA for the TAPSA model and (ii) TCPSA and TBPSA for the TCPSA model. For the third iterative stage (the multiplicative step that assessed whether combinations of ratios and/or zone sizes provided additional predictive information), combinations of the three top performing candidates from the preceding steps were assessed. For each candidate, the optimum test sensitivity, specificity, accuracy, positive likelihood ratio, and negative likelihood ratio from Youdon index-calculated optimum binary cutoff values were calculated.
